# Industry 4.0: A Proposal of Paradigm Organization Schemes from a Systematic Literature Review

**DOI:** 10.3390/s22010066

**Published:** 2021-12-23

**Authors:** Cristian Rocha-Jácome, Ramón González Carvajal, Fernando Muñoz Chavero, Esteban Guevara-Cabezas, Eduardo Hidalgo Fort

**Affiliations:** Department of Electronics Engineering, University of Seville, 41092 Seville, Spain; crjacome@us.es (C.R.-J.); fmunoz@us.es (F.M.C.); eguevara@us.es (E.G.-C.); ehidalgo@us.es (E.H.F.)

**Keywords:** Industry 4.0, cyber–physical system, IIoT, big data, cloud computing, digital twin, cyber security, artificial intelligent, digital maturity, blockchain

## Abstract

Currently, the concept of Industry 4.0 is well known; however, it is extremely complex, as it is constantly evolving and innovating. It includes the participation of many disciplines and areas of knowledge as well as the integration of many technologies, both mature and emerging, but working in collaboration and relying on their study and implementation under the novel criteria of Cyber–Physical Systems. This study starts with an exhaustive search for updated scientific information of which a bibliometric analysis is carried out with results presented in different tables and graphs. Subsequently, based on the qualitative analysis of the references, we present two proposals for the schematic analysis of Industry 4.0 that will help academia and companies to support digital transformation studies. The results will allow us to perform a simple alternative analysis of Industry 4.0 to understand the functions and scope of the integrating technologies to achieve a better collaboration of each area of knowledge and each professional, considering the potential and limitations of each one, supporting the planning of an appropriate strategy, especially in the management of human resources, for the successful execution of the digital transformation of the industry.

## 1. Introduction

In a globalized and highly competitive world, an industry that is not willing to innovate, to constantly improve its processes or to use emerging technologies may be condemning itself to disappear. That is why the concept of Industry 4.0 has become a fundamental pillar, both in current research and in its application in the industrial sector. There is a classification made by The IMD World Digital Competitiveness Ranking 2021, which can be a benchmark of the technological reality of industrialized countries. This ranking annually conducts studies that measure the capacity and readiness of 63 economies to adopt and explore digital technologies for economic and social transformation, considering factors, such as knowledge, technology, and preparation for the future [1].

The Industry 4.0 transition offers many opportunities to reinvent global supply chains with sustainability in mind by significantly improving supply chain processes and achieving strategic results. This digitization drive has become the mainstay of engineering organizations, regardless of size. Industry 4.0 will help organizations achieve sustainable growth and generate higher values in profits and results through faster design and development, innovative products, lower risk and reducing waste to the minimum amount possible. The social point of view reveals that technological modernization is expected to influence the spread of social transformation, especially in developing countries. However, its implementation faces technological and social challenges. It is important to identify contemporary trends and future prospects of sustainable Industry 4.0 and to study more rigorously its impact on sustainable development [2].

The stages in the development of industrial manufacturing systems, from manual labor to the concept of Industry 4.0, can be presented as a path through the four industrial revolutions. This path has been covered in many scientific papers; among them, we can mention [3,4]. The First Industrial Revolution began in the late 18th and early 19th century and was characterized by the introduction of mechanical manufacturing systems, using water and steam. The Second Industrial Revolution began in the late 19th century, symbolized by mass production based on the use of electric power. The Third Industrial Revolution began in the mid-20th century and introduced automation and microelectronic technology in manufacturing. Today, we are in the fourth industrial revolution driven by the development of information and communication technologies (ICT). Its technological basis is the intelligent automation of cyber–physical systems with decentralized control and advanced connectivity (IoT functionalities) [5,6]. The consequence of this new technology for industrial production systems is the reorganization of classical hierarchical automation systems to a self-organizing cyber–physical production system that enables customized and flexible mass production in production quantity [7,8].

The human factor is fundamental in Industry 4.0; its application translates into the “redefinition” of jobs, avoiding isolated knowledge and ensuring interconnectivity, collaboration, and knowledge sharing [9,10]. Therefore, it is necessary to talk about computer supported collaborative work (CSCW) in a way that includes the processes and resources that are involved and offers an integration of tools and methods that support the collaboration of work teams and can potentially improve the productivity and effectiveness of those who work collaboratively [11,12]. CSCW is generally structured around four disciplines: psychological, sociological, organizational and technological. For this paper, we focus only on the technological theme.

We cannot talk about specific technologies, as the concept allows a constant evolution and a continuous integration of new technologies as they mature or adapt, i.e., several emerging technologies are converging to provide more and more digital solutions to the industry, but we will mention the main ones, and their application in this case study [13]. According to the authors of the papers [14,15], they clearly mention the technologies involved in the concept of Industry 4.0. They also propose in the paper [15] a conceptual framework, which they divide into front-end technologies and core technologies. The front-end technologies consider four dimensions (smart manufacturing, smart products, smart supply chain and smart work), while the base technologies consider four (Internet of Things, cloud services, big data and analytics). These works were of great help for our study, as they define a way to analyze the complex and multidisciplinary world of Industry 4.0; however, for our case, we will analyze in terms of paradigms with cyber–physical systems criteria. Our analysis proposal allowed us to clarify the concept and propose better roadmaps toward the fulfillment of each Industry 4.0 project objectives. Finally, the bibliometrics of the research is exposed in the most explicit and graphic way possible in order to not only deliver information to readers, but also as a motivation or inspiration for other researchers to have a vision of the opportunities presented by Industry 4.0 and digital transformation to see how world power countries invest in research in these areas and turn them into technological and economic leaders.

## 2. Materials and Methods

For the development of this work, we analyzed almost two hundred scientific documents including scientific articles, books, web pages and conference proceedings of the paradigms and main mature and emerging technologies that encompass the concept of Industry 4.0 to ease the understanding of its complexity.

We have tried to include as many of the technologies that are directly or indirectly involved in Industry 4.0 as possible in order to have a fairly broad and objective view of this concept. We mention the databases that were used for the collection of information: MDPI, IEEE, Taylor & Francis, Scopus, IET Inspect, dblp, EBSCO, DOAJ, Springer, Microsoft Academic, ULRICHS WEB, ELSEVIER, ScienceDirect, and Redalyc.

**Figure 1:**
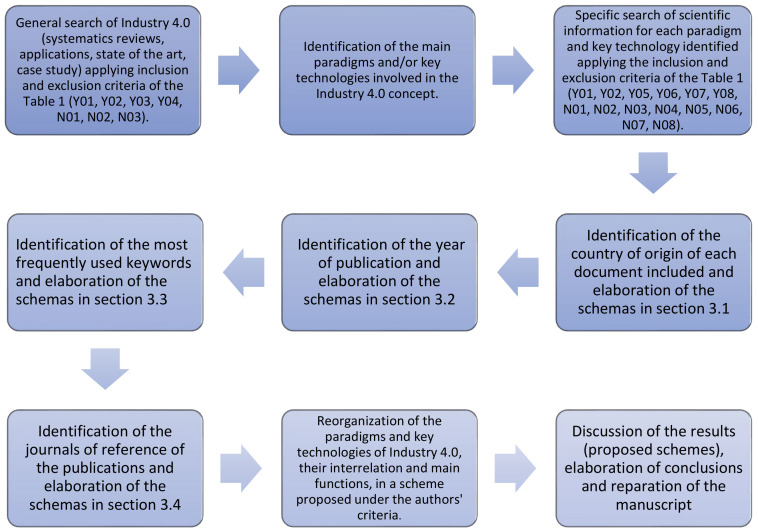
The main steps taken to validate the research.

This study follows the systematic review guideline and the Preferred Reporting Items for Systematic Reviews and Meta-Analyses (PRISMA) to ensure the soundness and reliability of the information used in this work. The main steps taken, including the protocols mentioned above, to validate our research are shown in Figure 1.

The research strategy started with an initial search in the aforementioned databases for scientific information with the following terms or keywords: “Industry 4.0”, “Smart factory”, “Fourth industrial Revolution”, “I4.0” and “Connected Industry 4.0”. The results of this first search allowed us to understand in a general way the concept of Industry 4.0, the studies that were developed on this topic and specifically to identify the main key technologies and paradigms involved in the Fourth Industrial Revolution. The inclusion/exclusion criteria from Table 1 applied were Y01, Y02, Y03, Y04, N01, N02, and N03. In addition, it is worth mentioning that no exclusions were applied due to the region, university or research center, and for this initial search, no restriction was applied with respect to the year of publication.

Once the key technologies and paradigms of Industry 4.0 were identified, the search for scientific information on each of them was individualized with a query as follows: “key technology” and “Industry 4.0”; “key technology” and “Smart factory”; “key technology” and “Fourth industrial Revolution”; “key technology” and “I4.0”, “key technology” and “Connected Industry 4.0”. This type of search was performed to ensure that the article studies the technology in question, but within the concept of Industry 4.0. The inclusion/exclusion criteria applied and present in Table 1 were Y01, Y02, Y05, Y06, Y07, Y08, N01, N02, N03, N04, N05, N06, N07, and N08. It should be noted that there are very few exceptions of articles that did not meet the year inclusion criteria (Y06); however, the content was relevant enough to be included in this study.

Furthermore, this study contains a bibliometric analysis technique to provide details of scientific production through statistical methods. The bibliometric analysis allows us to quantitatively evaluate the articles and recognize measurable patterns of interest, such as the occurrence of keywords, the geographical distribution of articles, year of publication, leading journals and databases. On the other hand, we used a content-centric analysis to identify qualitatively patterns, concepts and interrelation between the paradigms and the key technologies of Industry 4.0.

Finally, this study offers two alternative schemes to analyze the complex concept of Industry 4.0 from a practical perspective to provide industries with a clearer vision of the resources needed for a successful digital transformation.

The search for information for the preparation of this manuscript began in early April 2021, with the aim of presenting some preliminary data at the “XVI International Multidisciplinary Congress of Science and Technology CIT2021” held in June, where it was accepted and successfully presented. After that, the search for specific information intensified until mid-October. The number of articles of each key technology and paradigm found, excluded and included, are mentioned in Table 2.

### 2.1. Inclusion and Exclusion Criteria

Table 1 shows the inclusion and exclusion criteria used in this study, following the PRISMA protocols and the methodology of a systematic literature review.

The criteria were coded in order to be more easily used and understood in the context of this manuscript. Basically, these criteria allow us to guarantee the trustworthiness of the scientific information, that the sources are rigorous and reliable, and that the information is useful for the purposes of this study.

### 2.2. Metadata Extraction for Analysis

The strategy applied for the extraction of metadata for subsequent analysis was simple and manual. As an article was included under the inclusion and exclusion criteria, the following data were extracted for each one: geographical origin (country), year of publication, keywords, journal, or scientific publisher where it was published, and language. All this information was stored and classified in Excel spreadsheets for subsequent analysis and elaboration of diagrams. The results of this analysis can be found in the Bibliometric Analysis section.

## 3. Bibliometric Analysis

All this information allowed us to clarify the evolution of this concept to better abstract it from the academy and to propose strategies for its better analysis and treatment, due to the multidisciplinarity and constant evolution of the concept. This concept is understood as a point of convergence of technologies that is in constant expansion, in parity with technological advances to which new techniques and technologies are progressively annexed.

### 3.1. Summary of Information Selection

**Figure 2:**
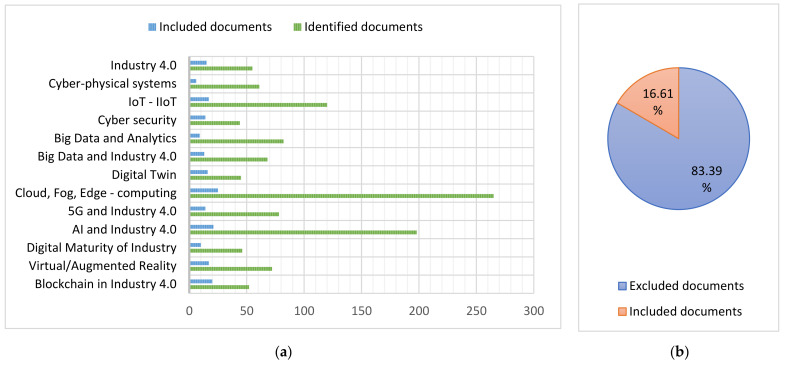
(**a**) Ratio of exclusion and inclusion of documents of each paradigm and key technology; (**b**) Percentage of documents included and excluded with respect to the total.

Table 2 summarizes the technologies, number of documents and their respective references analyzed for the elaboration of this work, the graphical representation of the data in this table is shown in Figure 2.

### 3.2. Geographical Distribution of Scientific Information

In Table 3, we identified the origin of each document used in this work in order to have a reference indicator of the countries that contribute scientifically to this topic. This indicator can give us a brief idea of the direct relationship between scientific research and the industrial development of each country.

**Figure 3:**
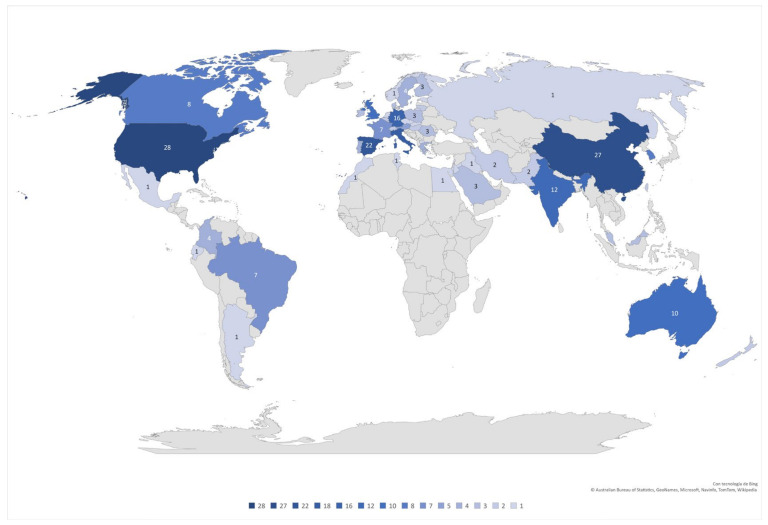
Geographical distribution and density of publications.

A map helps us to visualize geographically all the scientific contributions analyzed. It can be noticed that the United States and China are the countries that do the most research and contribute to this topic; in turn, we can suggest that they are the technologically, industrially, and economically dominant countries. It is shown in Figure 3.

**Figure 4:**
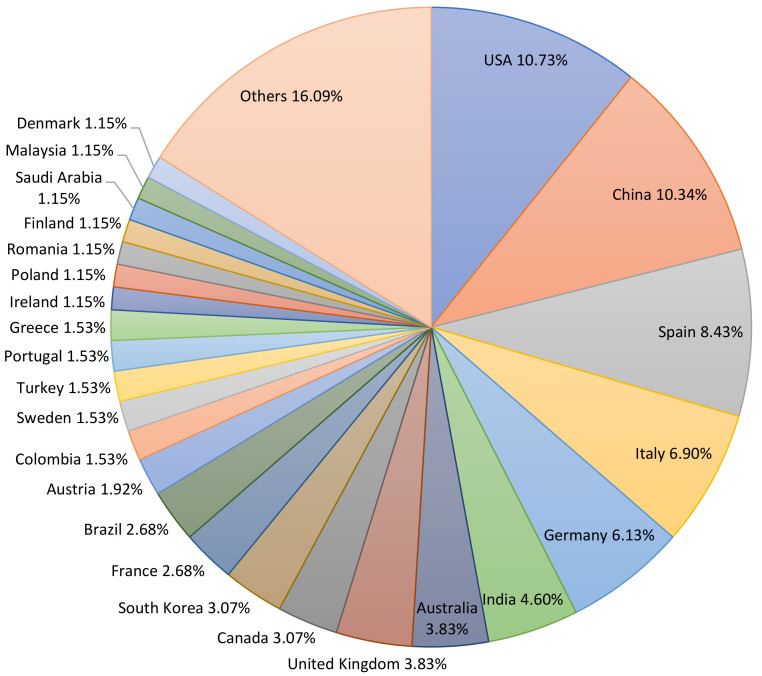
Percentage of countries’ contribution.

To find how much each country has contributed with its research to the elaboration of this study, each scientific paper was individualized, and its contribution was summed according to its authors and their affiliations. Each different affiliation per article was considered with a value of 1, represented as a percentage in Figure 4.

### 3.3. Chronology of Scientific Information

**Figure 5:**
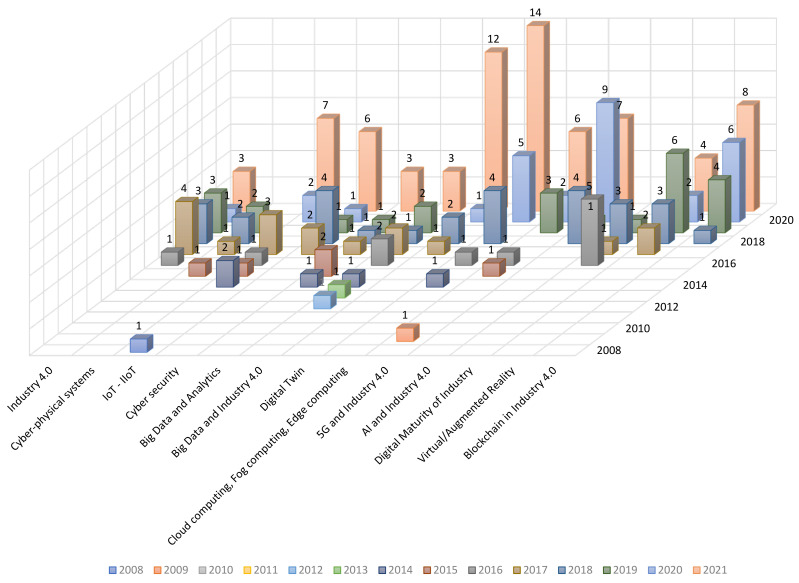
Chronology of scientific information on each technology and paradigm.

**Figure 6:**
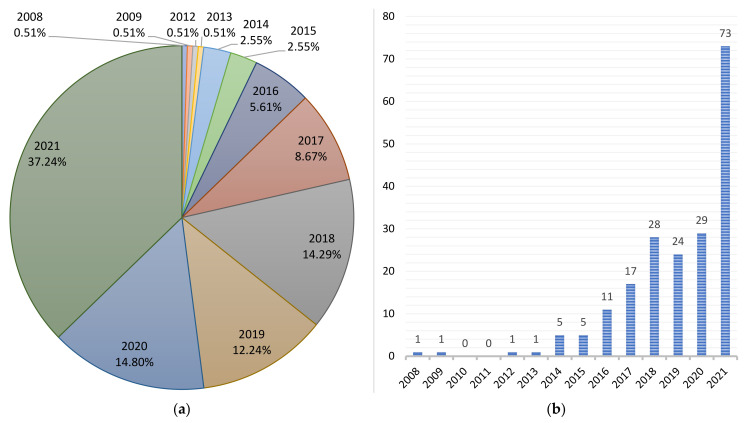
(**a**) Percentage ratio of scientific production by years; (**b**) distribution of scientific information by year.

Table 4 shows a chronological classification of the scientific papers according to the key technology analyzed. In addition, two schematic diagrams are presented in Figure 5 and Figure 6.

### 3.4. Keywords Most Frequently Used

**Figure 7:**
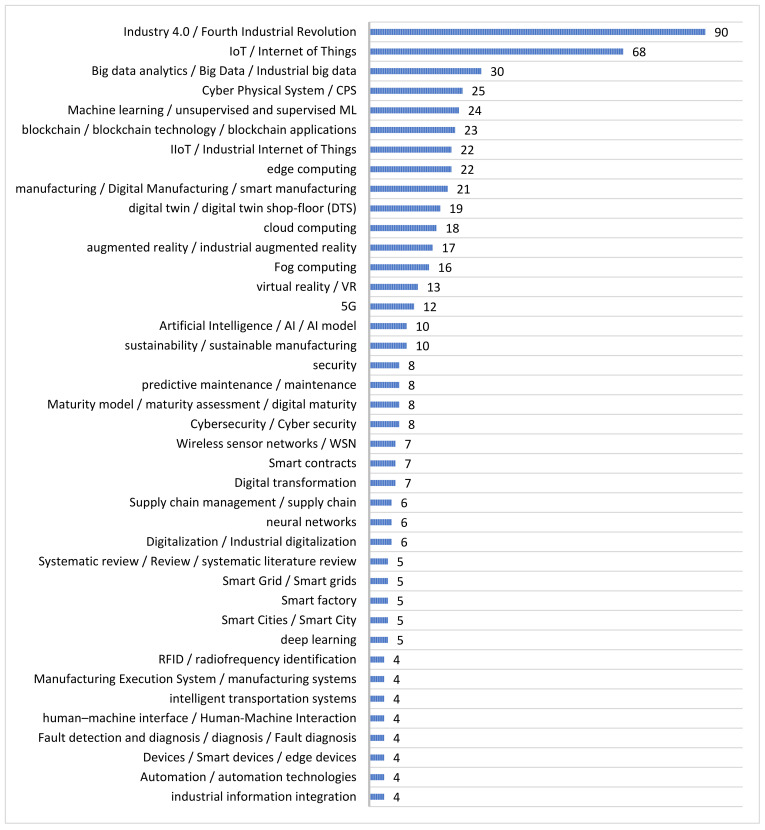
Keywords most frequently used.

A total of 457 different keywords were identified throughout the reference literature. Keywords with the same or very similar definitions or concepts were grouped together to obtain a better analysis of the frequency of occurrence of technologies or concepts within the Industry 4.0 study. In some scientific documents, in which there was no keyword section, nor was this information part of the file metadata, the main words of the title were considered the keywords of the document. Figure 7 shows the 40 most frequently occurring words.

### 3.5. Contribution Per Journal

**Figure 8:**
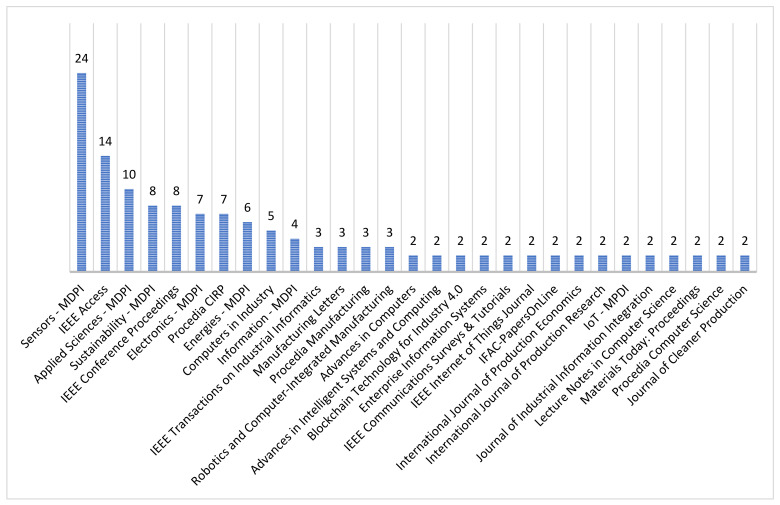
Leading journals in this work.

For this work, 87 different scientific sources were identified, including journals, books, book chapters and web pages. Figure 8 shows the journals that have contributed the most. The search for articles did not have criteria based on exclusive journals but were selected according to the topics and criteria mentioned in previous sections.

### 3.6. Language of References

**Figure 9:**
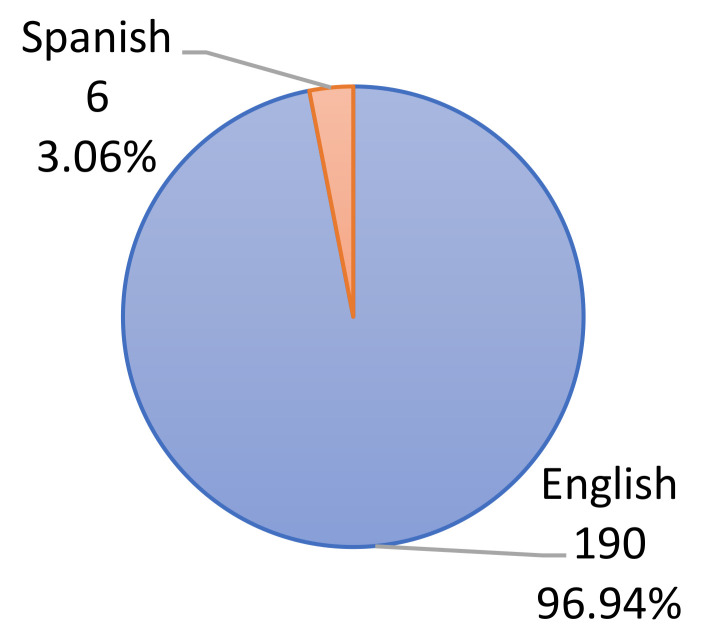
Language ratio.

Initially it was stated that the languages accepted would be English and Spanish, with preference always given to English. However, some interesting contributions in Spanish were found and considered. Figure 9 gives the language ratio of the total number of references consulted.

## 4. Results and Discussion

All the scientific information collected was understood and organized in two schemes, which we propose for an alternative and simple analysis of Industry 4.0.

A qualitative analysis of the information in the references was carried out to obtain the following schemes. These results were obtained by combining a descriptive and interpretative investigation of the references. The main phases of this analysis are as follows:

- Searching for and obtaining useful information.
- The preparation, review, and transcription of information.
- The organization of information and data according to criteria.
- The categorization, labeling and coding of information and data, which prepares them for analysis.
- The analysis of the data and the generation of propositions, usefulness, examples, and conclusions.

### 4.1. Criteria and Paradigms of Industry 4.0 Reorganized

Moving from strategy to execution is the key to an Industry 4.0 implementation study; however, having too much information about all the technologies involved in such a broad concept, this becomes a rather complicated task. Therefore, a poor execution will show us that the strategy, no matter how good it is, will not be successful and the desired objectives will not be achieved. That is why we make a proposal for the organization of I4.0 paradigms, technological knowledge areas; we place certain general aspects based on cyber–physical systems criteria, we highlight the importance of information and data, and finally we have a relationship with the classic pyramid of automation to better abstract the criteria with well-known theoretical concepts.

**Figure 10:**
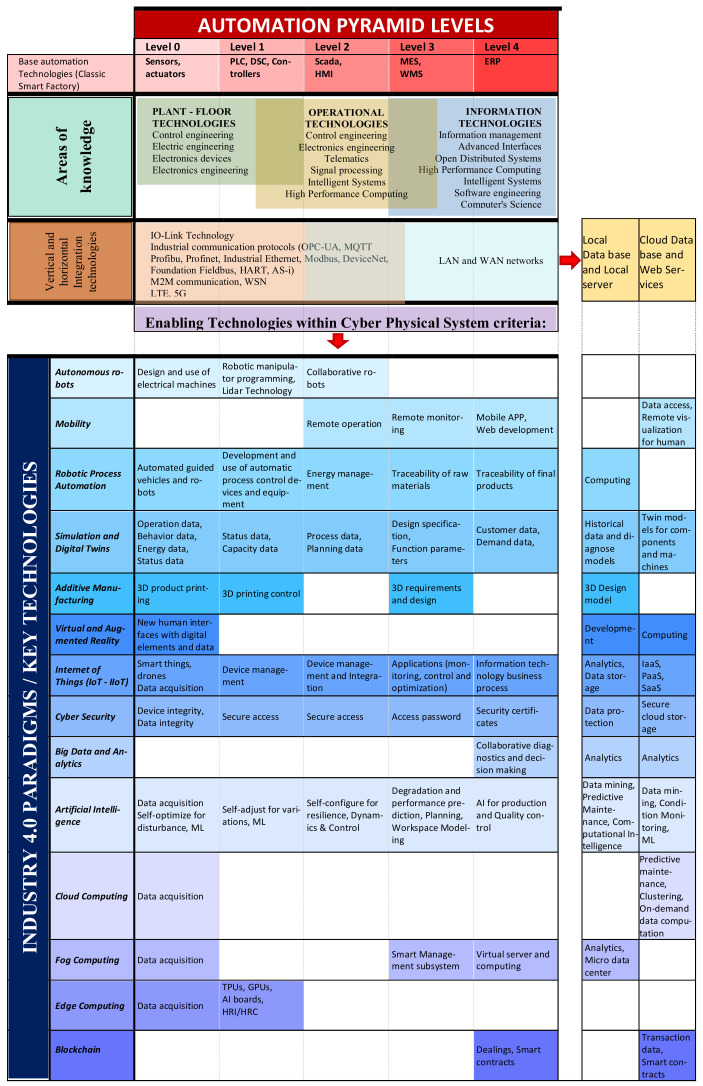
Proposed scheme of reorganization of Industry 4.0 criteria, paradigms, and key technologies.

This schematic in Figure 10 can help inspire and contribute to the design of strategic business plans that can be successfully implemented and align IT, OT and human resources to the execution of these plans. The scheme also serves another purpose, from the point of view of academia, to be analyzed and help the understanding of Industry 4.0 starting from better-known theoretical concepts.

Many general studies talk about the aspects involved in an industry in its digital transformation [1,2,3,4,5,6,7,8,9,10] and within the internal aspects of the value chain we can mention financial resources, social/brand capital, business processes, innovation capabilities, technology, human capital, organizational culture, and management capabilities. However, in this study, we focused on the technological aspects and their respective resources.

This first proposed scheme is based on the automation pyramid, a classification that is widely studied by levels in automation and smart factory issues so that we can start from a theoretical and standardized concept. Below, in our opinion, the areas of knowledge and the general technologies involved are placed in the scheme; this section can help us to plan an appropriate strategy, especially in the management of human resources and related professionals. The areas of knowledge give us a reference of the sciences in charge of the study or development of the key technologies and paradigms that are mentioned at the bottom of the scheme and are vertically aligned to the corresponding area of knowledge. In this way it is easier to identify the appropriate human resource according to the need for key technology that needs to be implemented or developed in a digital transformation of the industry.

Industry 4.0 is characterized by being an integral platform, so there are currently standardized protocols within IIoT of both vertical and horizontal integration that simplify these processes (for example, IO-LINK and MQTT). We can say to better understand that it would be enough a communication protocol to connect IoT devices with each other and at the same time with the cloud directly without the need to go climbing through the automation pyramid as it was analyzed before. That is why the vertical and horizontal integration is not treated with the same criteria as an automation pyramid, simply differentiating the two most notorious levels by aligning the areas of knowledge. We have mentioned the best-known communication protocols. From here on, information plays an important role.

Finally, we reorganized the paradigms of Industry 4.0, according to the areas of knowledge, related to the automation pyramid, and the participation of information within this concept, general aspects of the cyber–physical systems criteria were placed and where each of them would be participating.

In the left column, the key technologies and paradigm were placed, which are in bold and italics. In horizontal correspondence to each paradigm are placed the enabling or support technologies considering the cyber–physical systems criteria, each of these enabling technologies are at the same time vertically aligned with the knowledge areas and the automation pyramid. These enabling or supporting technologies are the more traditional, prevalent and in some cases mature technologies that allow the key technologies or paradigms of Industry 4.0 to perform their intended functions. This section of the scheme lists Industry 4.0 enabling technologies that we have recognized in the included articles. Many of these technologies have been available and commonly used in industry even for decades. In some literature, the prefix “smart” or “intelligent” is often used when naming these technologies, with the understanding that they are developed differently from the ground up to primarily support integration with each other.

The two columns on the right, slightly separated, try to explain the role of data in Industry 4.0. It starts from the vertical and horizontal integration, where its main function is the transmission of data, then briefly analyzes each key technology or paradigm to identify the role that each of them have in the management of both local data and in the cloud. These data and information management are one of the main characteristics of Industry 4.0, and practically intervene in almost all the paradigms analyzed; they could be considered core technologies of Industry 4.0 because they are an essential part of the integration between enabling technologies, key technologies and paradigms. Moreover, the treatment of this information and data in each paradigm is the basis of the Industry 4.0 concept.

Obviously not all technologies and criteria are present, and there will always be the possibility of integrating new technologies or new paradigms appearing. However, the proposed scheme is intended as a guide and reference to abstract in the best possible way the complex concept of Industry 4.0.

To better understand the scheme, we analyze some key technologies. Cybersecurity is a very important paradigm that is present at all levels of automation. Especially in the protection of data, both local and in the cloud, we can mention at lower levels that the role of cybersecurity is to ensure the integrity of the device and the integrity of the data obtained; the following levels must mainly ensure secure access to data and secure data transmissions, finally reaching the exclusive protection of local data and secure cloud storage. This key technology, as can be seen, is involved in all levels of Industry 4.0, and we can corroborate this by knowing that the core of the digitization of industry is the management of data at all levels, and these must always be protected.

Another key technology is cloud computing. This technology, despite being very important within Industry 4.0, is exclusively involved in data collection, data transmission to the cloud, and data computation in the cloud; in other words, it is not present at all levels.

IoT and IIoT have functions that are present at all levels of both automation and data, require professionals from all technological areas and are mentioned in the scheme. This undoubtedly ratifies the importance of this key technology in Industry 4.0; it is the fundamental pillar that supports, even with its architecture, the basis of cyber–physical systems.

### 4.2. CSCW Matrix for Industry 4.0 Paradigms

Our proposal stems from the initial difficulty of being able to place an Industry 4.0 paradigm in a single specific quadrant of the classic CSCW matrix, obviously because the CSCW concept is very useful but relatively old with respect to these emerging technologies. The matrix has had certain modifications to adapt as best as possible to the collaborative participation of each paradigm analyzed.

The purpose of this classification is to understand that these new concepts are not so much spatially or temporally located in a single quadrant, but that their scope goes beyond these classic boundaries and that they are collaborative and interdependent. For example, augmented reality interacts directly with the plant and machinery (same place) but requires high computational capacity and processing speed that is done with cloud computing (different place) all working in real time (same time), being a paradigm that is difficult to place in a classic automation pyramid or a classic CSCW matrix. However, there are also paradigms that can be placed in a single quadrant, as is the case of edge computing, which is characterized by performing computing and data storage in the location where it is needed (same place) to improve response times (same time) and save bandwidth.

Additionally, we considered adding a level to the place axis. Initially, in a CSCW matrix, there are only “same place” and “different place”, but in some paradigms, they can be interpreted to work in intermediate places, which we call “near place”, as is the case of fog Computing, which is part of a decentralized architecture where an additional edge processing unit is used to perform a substantial amount of computation, storage and communication locally not precisely on the device, but as an aid to cloud computing because it reduces the traffic between IoT devices and the cloud, i.e., for data to be processed locally and devices to communicate with each other, an additional processing unit has to be added to the IoT devices.

**Figure 11:**
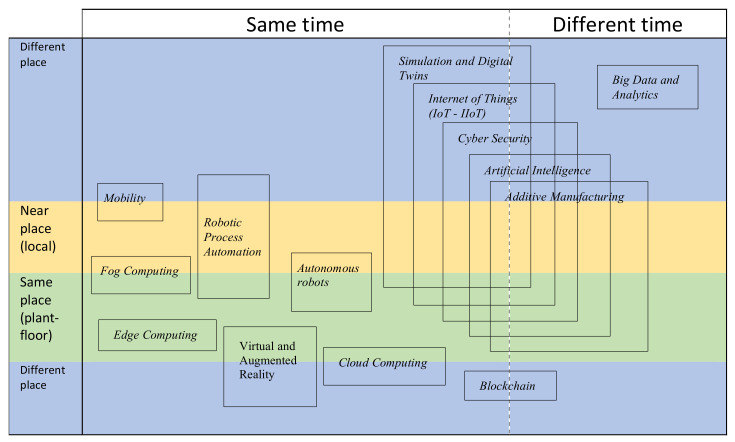
Proposed CSCW matrix for Industry 4.0 paradigms and key technologies.

Subsequently, the rest of the paradigms were placed according to their functions. Figure 11 shows a CSCW matrix adapted to the Industry 4.0 concept.

## 5. Conclusions

The proposed Industry 4.0 paradigm organization schemes will help academia and the industrial sector with little experience to propose more appropriate strategies according to the needs, available resources, and resources that will be required to ensure a successful implementation.

There is always the possibility that new technologies, new criteria, and new paradigms can be integrated into the proposed schemes, or even be modified due to the different opinions of other professionals in certain technical aspects, because Industry 4.0 is a rather broad topic, and it is also constantly evolving along with the rapid technological progress.

Moving from strategic planning to successful execution requires not only a correct digital maturity assessment and a proper roadmap, but also the proper management of resources: IT, OT, and human resources. Being as clear as possible about the complex concept of Industry 4.0 and its paradigms is very important for human resource management and related professionals to implement the successful execution of these plans.

The proposed schemes are based on an exhaustive literature search, but also on criteria and experience from an ongoing project based on the needs and problems that have to be faced; therefore, relatively different criteria may exist. The main purpose is to be a classification basis to better understand the Industry 4.0 concept.

The large amount of scientific information on these topics is a limiting factor for a more complete analysis, so there are still technologies that were not mentioned and that can be included in this proposed scheme. However, the main ones are found in the schemes.

Global market conditions have been strongly influenced by the COVID-19 pandemic and have led us to understand the importance of technology in the industry. This has led many companies to envision an early digital transformation, which will require as much information as possible to assist in the execution of their strategies.

Industry 4.0 paradigms have a spatial and temporal distribution that do not fit in a classical CSCW matrix, so it is necessary to adapt a matrix to best represent this new form of distribution. This is due to the integration of several technologies that become more complex concepts and difficult to be abstracted in a traditional way.

Investment in research on these topics has a direct influence on the economy of each country; countries that do more research on these topics are those that are more advanced industrially, and it does not seem to be a casual coincidence of this direct relationship of the documents analyzed in this study. Therefore, it is necessary to encourage research in sectors with developing economies.

Today, companies are strongly considering the option of digital transformation and entry into Industry 4.0, and thus, the scope, and technology trends for sustainable results. Industry 4.0 offers different solutions for sustainability, including contributing to social sustainability that provides a good working environment that is safer and more pleasant for workers.

In spite of being a widely studied topic and having a lot of scientific information, the amount of information is not enough to understand the interrelation of the technologies that integrate the concept of Industry 4.0, which became an obstacle at the moment of applying the concepts and above all for taking advantage of the available human resources. There is some conflict in the scope of each professional in different areas of knowledge, as well as the limitations of the same professionals. For example, professionals in the areas of IT and ICT experts in developing software applications, KPIs, APIs, etc., have limited knowledge in areas of electronics and automation; the same happens in the opposite case, that is, it would be impossible to work with only one type of professional. It is even difficult to work with each professional needed if they do not know their scope and limitations. That is why investigating the interrelationship of key technologies and paradigms of Industry 4.0, relating both areas of knowledge, levels of automation, information management, communications, with each paradigm and key technology, and representing them in a scheme was a necessity that now allows us to have a theoretical tool to continue with future research accompanied by real implementations in the industry.

## Figures and Tables

**Table 1:** Inclusion and exclusion criteria.

Category	Criteria	Code
Inclusion	The article corresponds to a scientific database and formally published.	Y01
	Articles completely written in English or Spanish	Y02
	The article addresses the topic of Industry 4.0 in a major way.	Y03
	The article mentions key paradigms and technologies involved in Industry 4.0 concept.	Y04
	The article relates the key technology to Industry 4.0.	Y05
	The article is updated no more than 5 years in the case of key technologies.	Y06
	The article details the contribution of the technology in question to Industry 4.0.	Y07
	If it is a web page, it must correspond to official scientific dissemination sites.	Y08
Exclusion	The article is not published in scientific databases and/or indexed journals.	N01
	The quality of the information is irrelevant for this study.	N02
	The information is redundant and of lower quality than previously included articles.	N03
	The article deals with emerging technologies but does not relate them to Industry 4.0.	N04
	The article deals with very particular case studies and with little detail.	N05
	The article mentions Industry 4.0 in its title or key words but does not address the topic in its content.	N06
	The information in the article is outdated with respect to others previously included.	N07
	If it is a web page, it corresponds to blogs or unofficial web pages.	N08

**Table 2:** Technologies and paradigms of Industry 4.0 analyzed.

Technology/Paradigm	Identified Documents	Excluded Documents	Included Documents	References
Industry 4.0	55	40	15	[1,2,3,4,5,6,7,8,9,10,11,12,13,14,15]
Cyber–Physical Systems	61	55	6	[16,17,18,19,20,21]
IoT–IIoT	120	103	17	[22,23,24,25,26,27,28,29,30,31,32,33,34,35,36,37,38]
Cyber security	44	30	14	[39,40,41,42,43,44,45,46,47,48,49,50,51,52]
Big Data and Analytics	82	73	9	[53,54,55,56,57,58,59,60,61]
Big Data in Industry 4.0	68	55	13	[62,63,64,65,66,67,68,69,70,71,72,73,74]
Digital Twin	45	29	16	[75,76,77,78,79,80,81,82,83,84,85,86,87,88,89,90]
Cloud, Fog, Edge computing	265	240	25	[91,92,93,94,95,96,97,98,99,100,101,102,103,104,105,106,107,108,109,110,111,112,113,114,115]
5G in Industry 4.0	78	64	14	[116,117,118,119,120,121,122,123,124,125,126,127,128,129]
AI in Industry 4.0	198	177	21	[130,131,132,133,134,135,136,137,138,139,140,141,142,143,144,145,146,147,148,149,150]
Digital Maturity of Industry	46	36	10	[151,152,153,154,155,156,157,158,159,160]
Virtual/Augmented Reality	72	55	17	[161,162,163,164,165,166,167,168,169,170,171,172,173,174,175,176,177]
Blockchain in Industry 4.0	52	32	20	[178,179,180,181,182,183,184,185,186,187,188,189,190,191,192,193,194,195,196]
Total	1186	989	197	

**Table 3:** Scientific contribution by country.

Country	Number of Documents
U.S.A.	28
China	27
Spain	22
Italy	18
Germany	16
India	12
Australia	10
United Kingdom	10
Canada, South Korea	8
France	7
Brazil	7
Austria	5
Colombia, Sweden, Turkey, Portugal, Greece	4
Ireland, Poland, Romania, Finland, Saudi Arabia, Malaysia, Denmark	3
Switzerland, Lithuania, Taiwan, Singapore, Pakistan, Iran, Belgium, New Zealand, Hungary	2
Slovakia, Ecuador, Russia, Norway, México, Morocco, Egypt, Estonia, Argentina, Macedonia, Malta, Czech Republic, the Netherlands, Jordan, Seoul, Israel, Mexico, Palestine, Lebanon, Tunisia, Iraq	1

**Table 4:** Technologies and paradigms publication year.

Technology/Paradigm	2008	2009	2012	2013	2014	2015	2016	2017	2018	2019	2020	2021
Industry 4.0							1	4	3	3	1	3
Cyber–Physical Systems						1		1	2	2		
IoT–IIoT	1				2	1	1	3			2	7
Cyber security								2	4	1	1	6
Big Data and Analytics					1	2		1	1	1		3
Big Data and Industry 4.0			1	1	1		2	2	1	2		3
Digital Twin								1	2		1	12
Cloud computing, Fog computing, Edge computing					1		1		4		5	14
5G and Industry 4.0		1				1	1			3	2	6
AI and Industry 4.0									4	1	9	7
Digital Maturity of Industry							5	1	3	1		
Virtual/Augmented Reality								2	3	6	2	4
Blockchain in Industry 4.0									1	4	6	8

## Data Availability

Not applicable.
